# Cerebral Salt Wasting Syndrome following Head Injury in a Child Managed Successfully with Fludrocortisone

**DOI:** 10.1155/2016/6937465

**Published:** 2016-04-24

**Authors:** Nagendra Chaudhary, Santosh Pathak, Murli Manohar Gupta, Nikhil Agrawal

**Affiliations:** Department of Pediatrics, Universal College of Medical Sciences, Bhairahawa 32900, Nepal

## Abstract

Cerebral salt wasting (CSW) syndrome is an important cause of hyponatremia in head injuries apart from syndrome of inappropriate antidiuretic hormone (SIADH). Proper diagnosis and differentiation between these two entities are necessary for management as the treatment is quite opposite in both conditions. Fludrocortisone can help in managing CSW where alone saline infusion does not work. We report a 17-month-old female child with head injury managed successfully with saline infusion and fludrocortisone.

## 1. Introduction

Hyponatremia, a frequently occurring electrolyte disorder in critically ill neurological patients [1], especially traumatic brain injury, if not managed properly can lead to increase in mortality and morbidity [2]. SIADH and CSW are the two common causes of hyponatremia in such patients. The incidence of CSW in brain injury ranges from 0.8 to 34.6% [3]. SIADH is characterized by inappropriate retention of free water due to increased antidiuretic hormone (ADH) secretion while CSW results in polyuria and natriuresis leading to extracellular volume contraction [4]. It is important to differentiate between both, as SIADH requires fluid restriction while CSW needs saline infusion [5]. A mineralocorticoid, fludrocortisone, has also proven to be beneficial in cases of CSW.

We report a 17-month-old female child with head injury and CSW managed with fludrocortisone which is probably the first case reported from Nepal to the best of our knowledge.

## 2. Case Presentation

A 17-month-old female child was brought to the casualty with alleged history of fall from 2-storey (about 20 feet) building. She had swelling of right side of head with right ear bleeding. On examination, weight was 7.5 kg, pulse rate 110/min, respiratory rate 32/min, SPO_2_ 98%, blood pressure 90/60 mm of Hg, and temperature 98.2°F. Central nervous system examination revealed Glasgow coma scale (GCS): 10/15 (Eye opening-2, Motor response-4, Verbal-4), sluggishly reacting unequal pupils, increased tone, and extensor plantars. Child was kept nil per oral. She was started on maintenance intravenous fluids (IVF), phenytoin, dexamethasone, and antibiotics (ceftriaxone).

**Figure 1:**
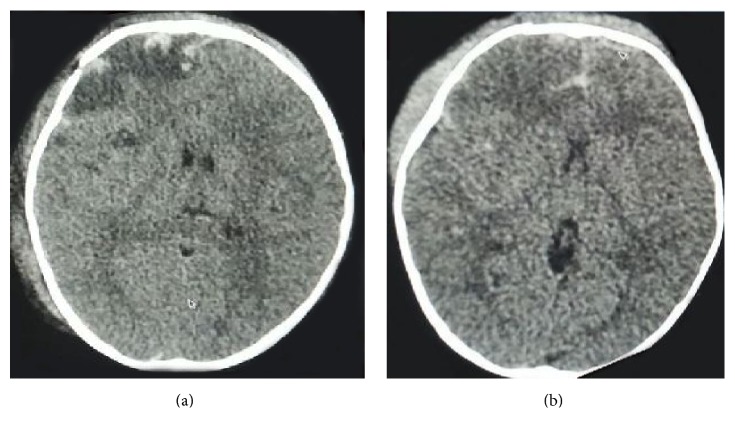
Computed tomography of head showing fracture of right frontal bone and subdural hematoma and extradural hematoma with contusion and subarachnoid hemorrhage.

Computed tomography of head was done which suggested fracture of skull bone with subdural hematoma, subarachnoid hemorrhage, extradural hematoma, and contusion (Figures 1(a) and 1(b)). Neurosurgical opinion was sought. Child had multiple episodes of seizures on day 3 of admission which was controlled with phenytoin (8 mg/kg/day), valproate (50 mg/kg/day), and levetiracetam (40 mg/kg/day).

**Figure 2:**
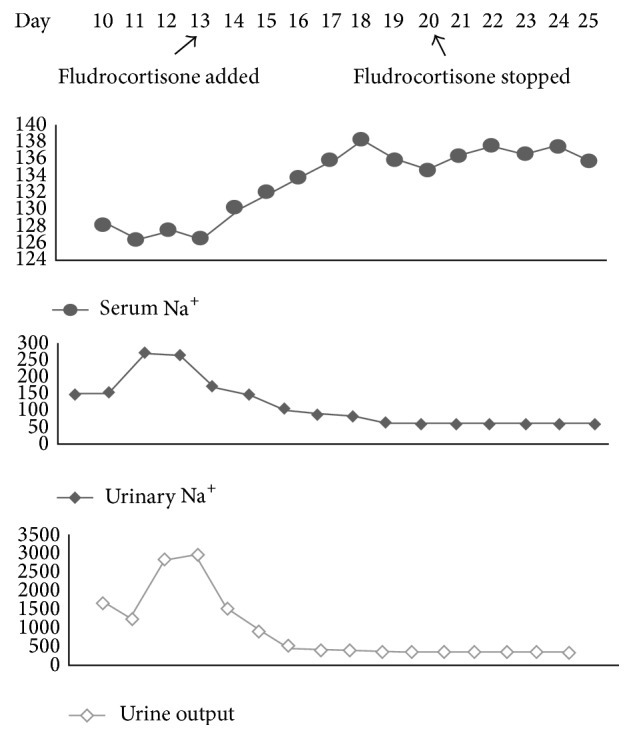
Relationship of urine output (mL/day), serum sodium (meq/L), and urinary sodium (meq/L) before and after treatment with fludrocortisone.

Child was then planned for oral feeding. Child gradually tolerated feeds well and intravenous fluid was stopped. The next day (day 10), child had polyuria (9 mL/kg/hr) with hyponatremia (128 meq/L). Blood urea was 22 mg/dL and serum creatinine was 0.6 mg/dL. Possibility of SIADH and CSW was considered. Urine osmolality and urine sodium were 400 mosmol/L and 146 meq/L, respectively, whereas serum osmolality was 265 mosmol/L. Serum uric acid was 3.5 mg/dL. Fractional excretion of sodium (4.16%) and uric acid (24.8%) was raised. Serum ADH level was within normal limits. She was diagnosed to have CSW as she had increased urinary Na^+^ excretion with polyuria and hyponatremia. Urinary replacement (volume to volume) with normal saline was done every 1-2 hours depending on the polyuria. Saline (DNS) infusion was given as maintenance fluid. As she still had hyponatremia, 3% NaCl infusion at 0.1 mL/kg/hr too was considered (maximum rate up to 0.3 mL/kg/hr) through central line (femoral line). Maximum amount of sodium infused was 41 meq/kg/day. Child's polyuria (reached up to 15 mL/kg/hr) was not controlled despite saline infusion. Child was then (day 13) started on oral fludrocortisone 0.1 mg/day, in two divided doses which increased to 0.2 mg/day after 2 days. Serum electrolytes and blood pressure monitoring were done regularly. Urine output improved gradually and polyuria subsided (Figure 2). Fludrocortisone was gradually tapered and stopped on day 20 of admission. Child also received two packed red blood cell transfusions during the admission period. Figure 2 shows the relationship of urine output, serum sodium, and urinary sodium concentration before and after treatment with fludrocortisone.

She was then started on oral feeding and intravenous fluid was gradually tapered. On discharge, child's sensorium was normal and she was on full feeds and had normal urine output and no seizures.

## 3. Discussion

CSW is characterized by hyponatremia and increased loss of sodium in urine with volume depletion and generally occurring in patients with head injuries [6], infections [7], or tumours. Hyponatremia is also common (30%) in patients with SAH. Peters et al. reported CSW as a salt wasting syndrome associated with cerebral disease in 1950s [8]. Some authors have reported that CSW is as common as SIADH [9]. The prevalence data in children is scarce with few case reports and case series [10, 11]. The postulated pathophysiology of CSW in head injury is (a) increased sympathetic activity and dopamine release causing natriuresis; (b) increased brain natriuretic peptide (BNP) by the injured brain; and (c) failure of renin aldosterone system [11, 12]. The treatments of hyponatremia in SIADH and CSW are completely opposite although both require increased salt intake. Improper diagnosis of the cause of hyponatremia may lead to inappropriate fluid restriction in CSW or fluid and salt supplementation in SIADH [13].

CSW is characterized by increased urinary sodium loss (increased FENa^+^), resulting in hyponatremia and finally extracellular fluid volume contraction whereas SIADH involves increased renal sensitivity to ADH or inappropriate ADH secretion which in turn leads to water conservation (dilutional hyponatremia) [2, 14]. Natriuretic peptides (ANP, BNP) are generally raised in CSW [15]. There is increased fractional excretion of uric acid in both CSW and SIADH, where the excretion improves in SIADH after correction but persists in CSW. BUN is generally raised in CSW and low in SIADH; however this is not universal in all cases. Inability to differentiate between them can lead to serious consequences. The salient differentiating points are given in Table 1.

Children with CSW generally have low blood pressure and even shock due to polyuria, increased urinary sodium loss, and contraction of extracellular fluid. Volume status determination remains difficult in such cases which requires CVP measurement [18]. Our patient also had low blood pressure which was managed with fluid boluses, maintenance intravenous fluid (5% DNS), and vasopressors (dopamine).

Fluid restriction is required in SIADH whereas saline infusion (0.9% NS/3% saline) is needed in treatment of CSW [11]. Although CSW should be treated aggressively, one should be cautious not to raise serum sodium >0.5 meq/L/hr to prevent pontine myelinolysis. Cases not responding alone to saline infusion should be treated with fludrocortisone, a synthetic mineralocorticoid.

The use of fludrocortisone in CSW has been reported in 1980s in adult patients with head injury [19, 20]. Data of its use in pediatric population with CSW has been reported sporadically [21–23]. Ozdemir et al. reported successful use of fludrocortisone in a 34-month-old child with lissencephaly who developed CSW after brain biopsy [17]. The duration of its use is variable. In a series of 4 cases reported by Taplin et al., fludrocortisone has been used for 4–125 days, with doses ranging from 0.2 to 0.4 mg/day [11]. Prolonged and high doses can lead to high blood pressure and sodium and water retention and hypokalemia [11].

## 4. Conclusion

CSW should always be considered whenever a clinician encounters hyponatremia in patients with cerebral disorders. One can diagnose CSW in hyponatremic patients by establishing contraction of extracellular volume. Increased urinary sodium and urate excretion and high urine osmolality and polyuria with elevated natriuretic peptides support the diagnosis. Fludrocortisone can be an effective drug in the management of CSW apart from saline infusion.

## Figures and Tables

**Table 1:** Differences between CSW and SIADH [16, 17].

Features	CSW	SIADH
Dehydration	Present	Absent
Serum sodium	Decreased	Decreased
Urinary sodium excretion	Increased	Variable
Urine osmolality	Increased	Increased
Serum osmolality	Low	Low
Vasopressin	Low	High
Polyuria	Present	Absent
BUN	Increased	Normal
Blood pressure	Low	Normal or increased
ANP	Increased	Normal
Treatment	Saline/3% NaCl	Fluid restriction
